# TLR2/caspase-5/Panx1 pathway mediates necrosis-induced NLRP3 inflammasome activation in macrophages during acute kidney injury

**DOI:** 10.1038/s41420-022-01032-2

**Published:** 2022-04-26

**Authors:** Chongbin Liu, Yanting Shen, Liuwei Huang, Jun Wang

**Affiliations:** 1 Clinical Research Center of Kidney Disease, State Key Laboratory of Organ Failure Research, Guangdong Provincial Institute of Nephrology, Guangdong Provincial Key Laboratory of Renal Failure Research, Division of Nephrology, Nanfang Hospital, Southern Medical University, Guangzhou, PR China; 2 Department of Nephrology, The First People’s Hospital of Kashi, Kashi, PR China

**Keywords:** Acute kidney injury, Cell death

## Abstract

Acute kidney injury (AKI) is characterized by necroinflammation formed by necrotic tubular epithelial cells (TECs) and interstitial inflammation. In necroinflammation, macrophages are key inflammatory cells and can be activated and polarized into proinflammatory macrophages. Membranous Toll-like receptors (TLRs) can cooperate with intracellular NOD-like receptor protein 3 (NLRP3) to recognize danger signals from necrotic TECs and activate proinflammatory macrophages by assembling NLRP3 inflammasome. However, the cooperation between TLRs and NLRP3 is still unclear. Using conditioned medium from necrotic TECs, we confirmed that necrotic TECs could release danger signals to activate NLRP3 inflammasome in macrophages. We further identified that necrotic TECs-induced NLRP3 inflammasome activation was dependent on ATP secretion via Pannexin-1 (Panx1) channel in macrophages. Next, we verified that TLR2 was required for the activation of Panx1 and NLRP3 in macrophages. Mechanistically, we indicated that caspase-5 mediated TLR2-induced Panx1 activation. In addition, we showed that necrotic TECs-induced activation of TLR2/caspase-5/Panx1 axis could be decreased in macrophages when TECs was protected by N-acetylcysteine (NAC). Overall, we demonstrate that danger signals from necrotic TECs could activate NLRP3 inflammasome in macrophages via TLR2/caspase-5/Panx1 axis during AKI.

## Introduction

Acute kidney injury (AKI) is a global public health problem; there are ~13.3 million AKI diagnoses and 1.7 million AKI-associated deaths annually [1]. Effective pharmaceutical therapy of AKI is still lacking [2]. To find effective pharmaceutical therapy, it is necessary to delineate the mechanism of AKI.

AKI is characterized by necrosis of tubular epithelial cells (TECs), which induces necroinflammation formed as an auto-amplification loop between necrotic TECs and interstitial inflammation [3, 4]. This loop can lead to exacerbated renal injury. Macrophages are key inflammatory cells participating the whole stage of AKI [5]. When stimulated by necrotic TECs, macrophages can be polarized into proinflammatory macrophages, which can promote a proinflammatory milieu to worsen the initial level of renal injury [6]. The recruitment of proinflammatory macrophages is dramatically increased within the first 48 h after ischemic AKI [7]. Depletion of macrophages before ischemic injury can protect TECs [7]. However, the mechanism of macrophages activation caused by necrotic TECs is still under investigation.

Necrotic TECs can release damage-associated molecular patterns (DAMPs) to activate macrophages and polarize macrophages [8]. Danger signals can be recognized by pattern recognition receptors (PRRs), which mainly include transmembrane expressed Toll-like receptors (TLRs) and intracellularly expressed nucleotide-binding oligomerization domain receptors (NLRs) [9]. Among NLRs, NOD-like receptor family, porin domain containing 3 (NLRP3) is specifically involved in DAMP recognition [10]. When activated, NLRP3 can recruit ASC and caspase-1 to form NLRP3 inflammasome complex and release IL-1β [11]. IL-1β, a hallmark of proinflammatory macrophages, can provoke further activation of macrophages and amplification of sterile inflammation [12]. Therefore, blocking NLRP3 activation might inhibit the activation of macrophages and decrease the necroinflammation in the early stage of AKI

TLR can coordinate with NLRP3 to deal with danger signals released by necrotic cells [9]. Of various TLRs, TLR2 is the major receptor in recognizing danger signals from necrotic cells [13]. A previous study has identified that necrotic cells can stimulate TLR2/NF-κB pathway [14]. And NF-κB signaling can prime NLRP3 inflammasome by upregulating the expression of NLRP3 and pro-IL-1β [15]. But, as shown by several studies, TLR2 signaling may directly activate NLRP3 and trigger assembly of the inflammasome complex [16–18]. It is thus intriguing to propose that necrotic TECs can cause assembly of NLRP3 inflammasome in macrophages via TLR2 signaling pathway, which might extend our understanding of the mechanism of necrosis-induced NLRP3 inflammasome activation in macrophages.

To investigate this hypothesis, we confirmed the activation of NLRP3 inflammasome in macrophages by necrotic TECs firstly. Then we showed that necrotic TECs-induced NLRP3 inflammasome activation was dependent on ATP secretion via Pannexin-1 (Panx1) channel in macrophages. Next, we verified that TLR2 was required for the activation of Panx1 and NLRP3 in macrophages. Mechanistically, we identified that caspase-5 mediated TLR2-induced Panx1 activation. Our findings suggest that TLR2/caspase-5/Panx1 axis might be a novel pathway of necrosis-induced NLRP3 inflammasome activation in macrophages.

## Results

### Macrophage infiltration accompanies with necrotic renal tubules in AKI murine model

**Fig. 1:**
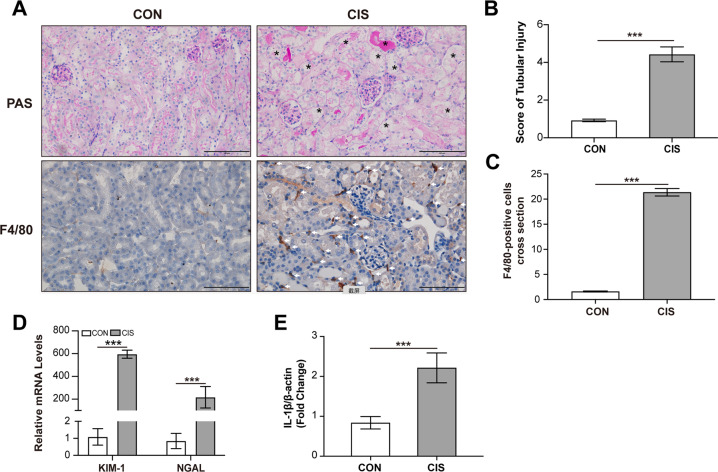
Macrophage infiltration increases in renal interstitium in cisplatin-induced AKI murine model. C57/BL mice were administered by intraperitoneal injection of cisplatin (25 mg/kg) and sacrificed 3 days later. Representative PAS staining images and immunohistochemical images F4/80 staining. Asterisk indicates injured renal tubules and white arrow indicates infiltrated macrophages. (scale bar, 100 μm) (**A**). Tubular injury score assessed in each group (**B**). Quantification of F4/80-positive cells by digital image analysis (**C**). Relative mRNA expressions of KIM-1 and NGAL in the renal tissue (**D**). Relative mRNA expressions of IL-1β in the renal tissue (**E**). Data are presented as the mean ± SD. *n* = 6 mice per group. The *p* values were calculated using Students *t* test. ***p < 0.001.

To clarify the distribution of macrophages in renal tissue post-acute injury, we constructed cisplatin-induced AKI murine model. Serum creatinine and BUN levels were significantly increased 72 h after cisplatin administration (Supplementary Fig. 1). PAS staining revealed injured renal tubules including the detachment of renal tubules and brush border damage and F4/80 immunohistochemical staining showed the increased infiltration of macrophages in the renal tissue of cisplatin-treated mice (Fig. 1A). Quantification of the tubular injury score was calculated to verify tubular necrosis induced by cisplatin (Fig. 1B). By quantitative summary of F4/80-positive cells per field, we confirmed an increased infiltration of macrophages in the renal tissue 72 h post cisplatin treatment (Fig. 1C). The mRNA levels of KIM-1 and NGAL in renal tissue were increased (Fig. 1D). The mRNA level of IL-1β was also increased in the renal tissue of cisplatin-treated mice (Fig. 1E). Therefore, these results indicate the increased macrophage infiltration around necrotic renal tubules in the early stage of AKI.

### DAMPs from necrotic TECs can activate NLRP3 inflammasome in macrophages

The induction of necrosis in TECs was performed by treating TECs with various concentrations of cisplatin. We verified the viability of TECs by using cell counting. Flow cytometry method evaluated the percentage of necrotic TECs by analyzing cells stained with Annexin V and 7-AAD. Primary necrotic cells were Annexin V^−^ and 7-AAD^+^ and secondary necrotic cells were Annexin V^+^ and 7-AAD^+^. We detected over 50% of TECs were necrotic when TECs were treated with 20 μM cisplatin for 6 h and harvested after 48 h (Supplementary Fig. 2).

**Fig. 2:**
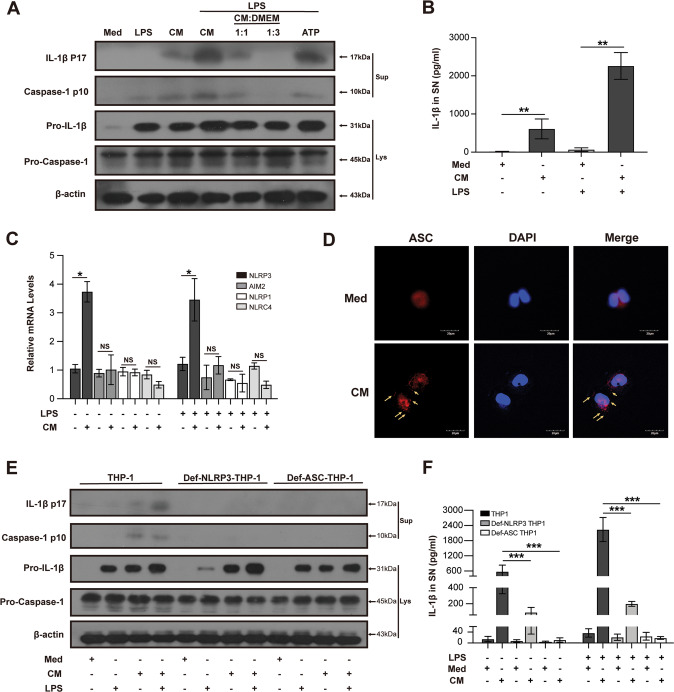
Necrotic TECs activate NLRP3 inflammasome in macrophages. PMA-differentiated THP-1 cells were treated with or without 100 ng/ml LPS for 3 h before incubated with different concentrations of conditioned medium (CM, primary concentration; CM 1:1, 1:1 dilution with culture medium; CM 1:2, 1:2 dilution with culture medium) for 6 h, incubated with 5 mM ATP for 1 h (positive control) or incubated with medium from normal NRK-52E cells (Med) as control. Culture supernatants (Sup) and cell lysates (Lys) were collected. Representative immunoblot analyses of the immature (Pro-) and mature forms of caspase-1 and IL-1β in culture supernatants and cell lysates (**A**). Quantification of IL-1β in supernatant under different treatments by ELISA (**B**). Relative mRNA expressions of NLRP3, AIM2, NLRP1 and NLRC4 in THP-1 cells (**C**). Representative immunofluorescence images of ASC in THP-1 cells. Arrows: ASC specks (scale bar, 20 μm) (**D**). Representative immunoblot analysis of the immature (Pro-) and mature forms of caspase-1 and IL-1β in culture supernatants and cell lysates from THP-1 cells, Def-NLRP3-THP-1 cells, and Def-ASC-THP-1 cells (**E**). Quantification of IL-1β in supernatant of THP-1 cells under different treatments by ELISA (**F**). Data are presented as the mean ± SD of the representative experiment performed in at least three biological replicates. The *p* values were calculated using one-way ANOVA test. *p < 0.05; **p < 0.01; ***p < 0.001.

To examine whether necrotic TECs can activate NLRP3 inflammasome in macrophages, PMA-differentiated THP-1 macrophages were exposed to conditioned medium from necrotic TECs (CM) for 6 h. The collection of CM and control medium (Med) was illustrated in a pattern diagram (Supplementary Fig. 3). To magnify the effects of CM on macrophages, we treated macrophages with LPS before CM treatment. We detected that the protein levels of mature IL-1β and cleaved caspase-1 increased in the supernatant of THP-1 macrophages (Fig. 2A). This means that necrotic TECs could activate inflammasome in THP-1 macrophages. ELISA assay also detected the protein level of IL-1β in supernatant of THP-1 cells, which was corresponding with immunoblot results (Fig. 2B). We found the increased mRNA level of NLRP3 and did not detect the upregulation of other intracellular PRRs including AIM2, NLRP1 and NLRC4 in THP-1 macrophages (Fig. 2C). We next examined ASC oligomerization in macrophages, which is also a signal of NLPR3 inflammasome activation. Using immunofluorescence assay, we detected ASC oligomerization in THP-1 macrophages treated by CM (Fig. 2D). To confirm NLRP3 inflammasome activation in THP-1 macrophages, we used Def-NLRP3-THP-1 cells and Def-ASC-THP-1 cells to observe the effects of CM. Almost no cleaved caspase-1 and mature IL-1β could be detected in Def-NLRP3-THP-1 and Def-ASC-THP-1 cells challenged with CM (Fig. 2E). ELISA assay agreed with immunoblot results (Fig. 2F). Treatment with a pan-caspase inhibitor (Z-VAD) or a caspase-1-specific inhibitor (Z-YVAD) proved the role of caspase-1 in THP-1 macrophages treated by CM. Inhibition of caspases almost completely abolished CM-induced IL-1β release in THP-1 macrophages (Supplementary Fig. 4). Together, these data imply that NLRP3 inflammasome in macrophages can be activated by DAMPs from necrotic TECs.

### Panx1 mediates NLRP3 inflammasome activation in macrophages

**Fig. 3:**
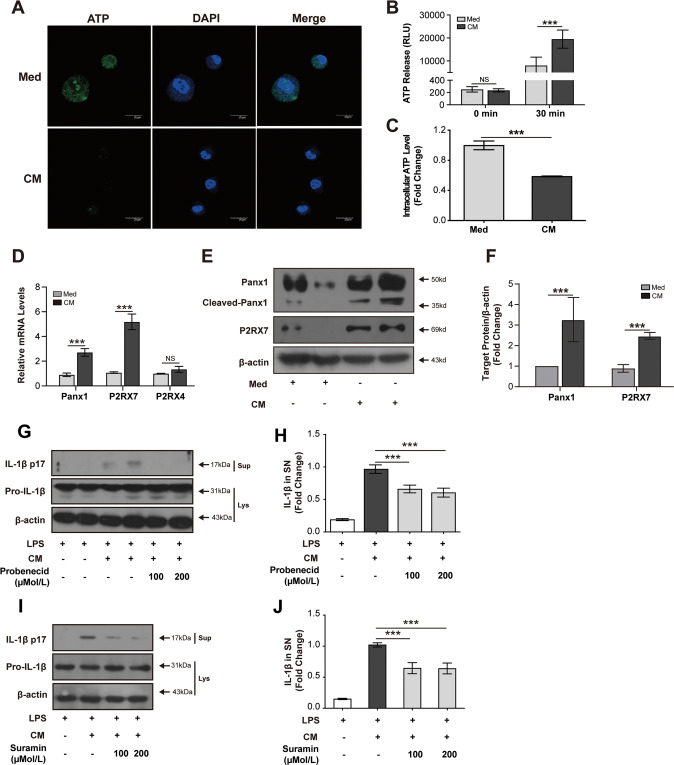
Panx1/ATP/P2RX7 axis is required for necrotic TECs-induced activation of NLRP3 inflammasome in macrophages. Representative immunofluorescence images of ATP in PMA-differentiated THP-1 cells (scale bar, 20 μm) (**A**). Quantification of ATP level in culture supernatant and in cell lysate of THP-1 cells by ATP assay kit (**B**, **C**). Relative mRNA expressions of Panx1, P2RX4, and P2RX7 in THP-1 cells (**D**). Representative and quantitative immunoblot analyses of Panx1 and P2RX7 (**E**, **F**). PMA-differentiated THP-1 cells were pretreated with probenecid (100 μM, 200 μM) or suramin (100 μM, and 200 μM) for 1 h and further stimulated with CM for 6 h. Representative and quantitative immunoblot analyses of the immature and mature forms of IL-1β in culture supernatants and cell lysates of THP-1 cells treated with probenecid or suramin (**G**, **I**). Quantification of IL-1β in supernatant of THP-1 cells treated with probenecid or suramin by ELISA (**H**, **J**). Data are presented as the mean ± SD of the representative experiment performed in at least three biological replicates. The *p* values were calculated using Students *t* test or one-way ANOVA test. *p < 0.05; **p < 0.01; ***p < 0.001.

Having demonstrated the activation of NLRP3 inflammasome in macrophages by necrotic TECs, we further investigated the molecular mechanism for this activation. Given that Panx1-mediated ATP excretion is a well-studied mechanism of NLRP3 inflammasome activation [19], we addressed whether Panx1 is involved in NLRP3 inflammasome activation in macrophages treated by necrotic TECs. Immunofluorescence staining and intracellular ATP detection showed that intracellular ATP was decreased in THP-1 macrophages treated with CM (Fig. 3A, C). ATP detection assay showed that extracellular ATP concentration increased obviously in THP-1 cells treated by CM for 30 min (Fig. 3B). CM treatment could upregulate the mRNA levels of P2RX7 and Panx1 in CM-treated THP-1 macrophages (Fig. 3D). Immunoblot also showed the increased protein levels of cleaved Panx1 and P2RX7 in CM-treated THP-1 macrophages (Fig. 3E, F). To further prove the role of Panx1 in NLRP3 inflammasome activation, we interfered with this axis with Panx1 channel blocker (probenecid) or P2RX7 antagonist (suramin). Decreased IL-1β production in CM-treated THP-1 macrophages could be observed under these interventions (Fig. 3G–J). Overall, these results indicate that Panx1 is required for NLRP3 inflammasome activation in macrophages stimulated by necrotic TECs.

### TLR2 is required for Panx1 cleavage and NLRP3 inflammasome activation in macrophages

**Fig. 4:**
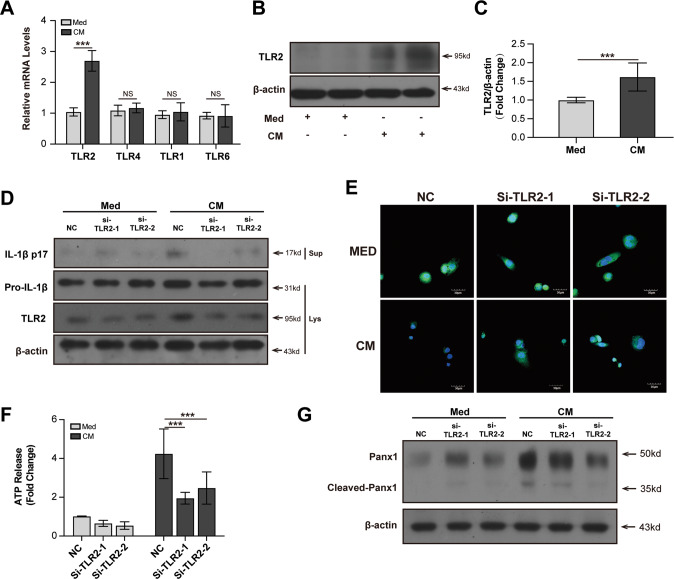
TLR2 is required for Panx1 cleavage and NLRP3 inflammasome activation in macrophages stimulated by necrotic TECs. Relative mRNA expressions of TLR1, TLR2, TLR4, and TLR6 in THP-1 cells (**A**). Representative and quantitative immunoblot analysis of TLR2 in THP-1 cells (**B**, **C**). PMA-differentiated THP-1 cells were transfected with TLR2 siRNA for 36 h before incubated with CM. Representative and quantitative immunoblot analysis of the immature and mature forms of IL-1β in supernatant and cell lysates of THP-1 cells (**D**). Representative immunostaining images of ATP in THP-1 cells (scale bar, 30 μm) (**E**). Quantification of ATP in culture supernatant of THP-1 cells by ATP assay (**F**). Representative and quantitative immunoblot analyses of Panx1 and cleaved Panx1 in cell lysates of THP-1 cells (**G**). NC Negative control siRNA. Data are presented as the mean ± SD of the representative experiment performed in at least three biological replicates. The *p* values were calculated using Students *t* test or one-way ANOVA test. *p < 0.05; **p < 0.01; ***p < 0.001.

A previous study showed that TLR stimulation can induce NLRP3 inflammasome activation via Panx1/ATP/P2RX7 axis [20], so we detected the expression of various TLRs in CM-treated THP-1 macrophages. We found that both the mRNA and protein levels of TLR2 were upregulated in CM-stimulated THP-1 macrophages. The mRNA levels of TLR1, TLR4, and TLR6 were not changed in THP-1 macrophages (Fig. 4A–C). Knockdown of TLR2 could decrease the protein levels of IL-1β in CM-treated THP-1 macrophages (Fig. 4D). Immunofluorescence staining of ATP showed that knockdown of TLR2 could increase the intracellular ATP expression in THP-1 macrophages (Fig. 4E). ATP secretion was also reduced in CM-treated THP-1 macrophages by knocking down TLR2 (Fig. 4F). Knocking down TLR2 could also decrease the protein level of cleaved Panx1 in CM-treated THP-1 macrophages (Fig. 4G). To verify the specific effects of TLR2 on ATP secretion, we also knocked down TLR4 in THP-1 macrophages and did not detect a significant decrease in ATP released in CM-treated THP-1 macrophages (Supplementary Fig. 5). Together, these results show that DAMPs from necrotic TECs can stimulate TLR2 to activate Panx1 and NLRP3 inflammasome in macrophages.

### Caspase-5 mediates TLR2-induced Panx1 cleavage in macrophages

**Fig. 5:**
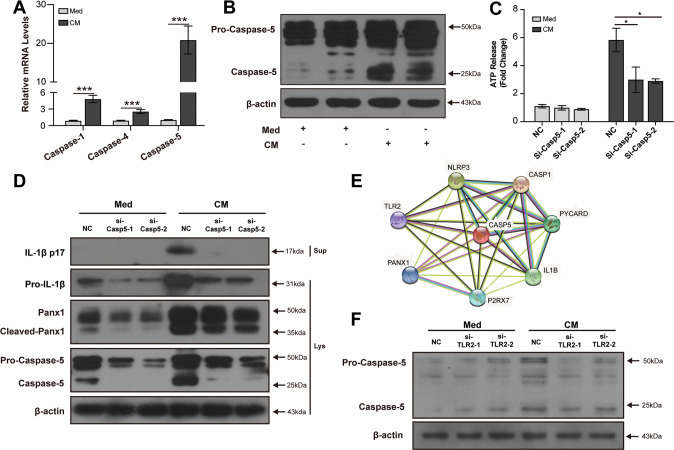
Caspase-5 mediates TLR2-induced Panx1 cleavage in macrophages. Relative mRNA expressions of caspase-1, caspase-4, and caspase-5 in THP-1 cells (**A**). Representative and quantitative immunoblot analysis of caspase-5 in cell lysates of THP-1 cells (**B**). PMA-differentiated THP-1 cells were transfected with caspase-5 siRNA for 36 h before incubated with Med or CM. Quantification of ATP in culture supernatant of THP-1 cells by ATP assay (**C**). Representative immunoblot analyses of the immature and mature forms of IL-1β, Panx1 and cleaved Panx1, and caspase-5 in supernatant and cell lysates of THP-1 cells (**D**). Computational prediction of caspase-5 protein partners network using STRING database (https://string-db.org) (**E**). PMA-differentiated THP-1 cells were transfected with TLR2 siRNA for 36 h before incubated with Med or CM. Representative immunoblot analyses of the immature and mature forms of caspase-5 in cell lysates of THP-1 cells (**F**). NC Negative control siRNA. Data are presented as the mean ± SD of the representative experiment performed in at least three biological replicates. The *p* values were calculated using Students *t* test. *p < 0.05; **p < 0.01; ***p < 0.001.

As Panx1 can be activated by caspase-mediated cleavage at C-terminal inhibitory domain [21], we detected the mRNA levels of caspase-1, caspase-4, and caspase-5 in CM-treated THP-1 macrophages. We found that caspase-5 mRNA level was upregulated obviously (Fig. 5A). Immunoblot also showed the increased protein level of caspase-5 in THP-1 macrophages (Fig. 5B). The secretion of ATP was reduced in macrophages after knocking down caspase-5 (Fig. 5C). Knockdown of caspase-5 could decrease the protein levels of both cleaved Panx1 and mature IL-1β (Fig. 5D). Furthermore, we used the STRING database (http://string-db.org/cgi/imput) to identify proteins that might interact with caspase-5 and found that caspase-5 has protein interactions with TLR2 and Panx1 (Fig. 5E). This indicates that caspase-5 mediates an association between TLR2 and Panx1. By knocking down TLR2, we detected the decreased protein level of caspase-5 in CM-treated THP-1 macrophages, which supports the correlation between TLR2 and caspase-5 (Fig. 5F). Together, these results showed that caspase-5 could mediate TLR2-induced Panx1 cleavage and NLRP3 inflammasome activation in macrophages stimulated by DAMPs from necrotic TECs.

### Activation of TLR2/Panx1/NLRP3 axis decreases in macrophages treated by protected TECs

**Fig. 6:**
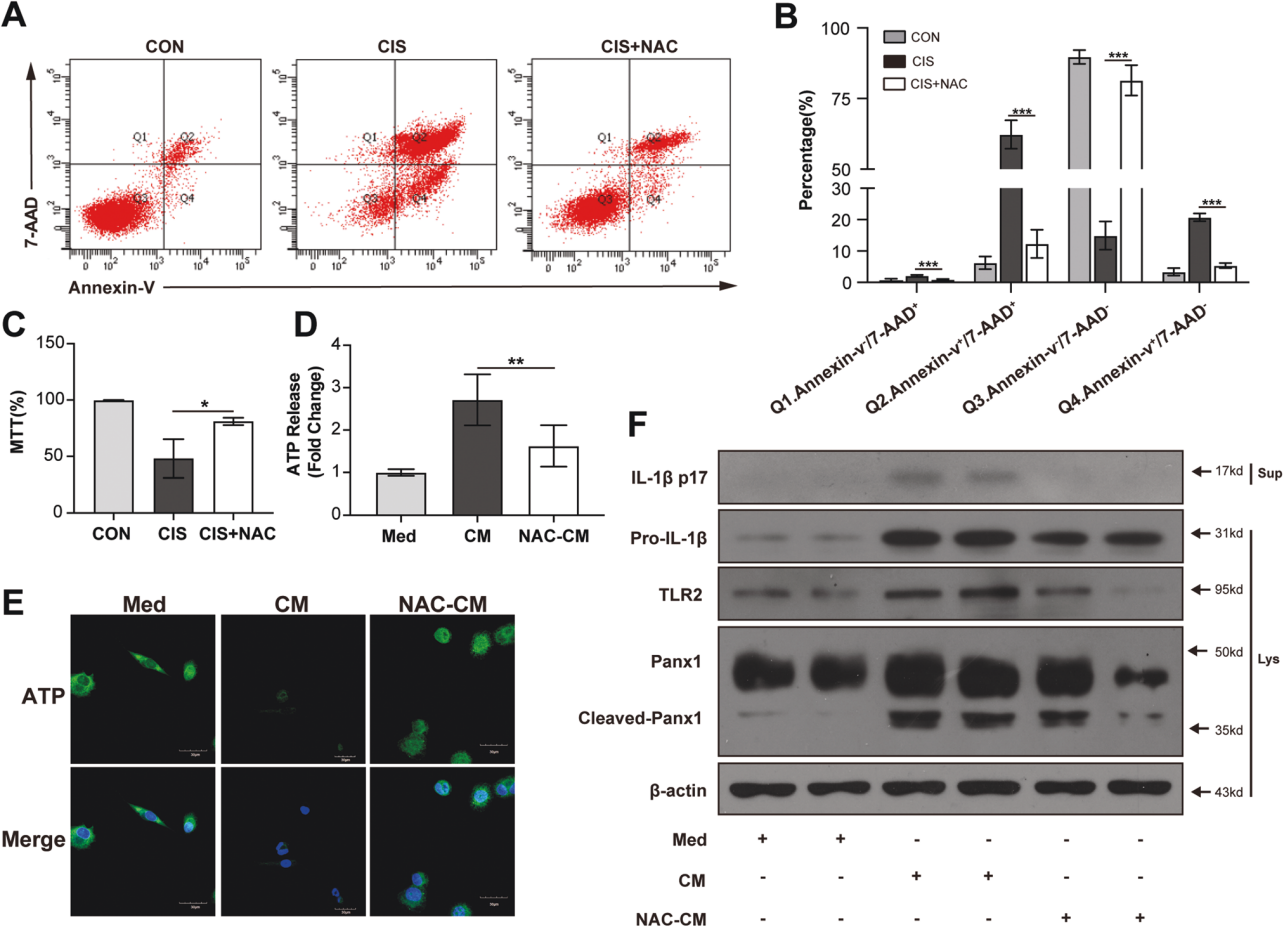
TLR2/Panx1/NLRP3 axis activation decreases in macrophages stimulated by NAC-protected TECs. NRK-52E cells were pre-incubated with 50 mM NAC before being treated with cisplatin and conditioned medium was collected (NAC-CM). Combined Annexin-V and 7-AAD staining was used to distinguish primary apoptotic (Annexin-V+/7-AAD−), primary necrotic (Annexin-V−/7-AAD+), secondary necrotic (Annexin-V+/7-AAD+), and live (Annexin-V−/7-AAD−) cells. Representative flow cytometry analysis and summarized results of TECs (**A**, **B**). The viability of TECs was analyzed by MTT assay (**C**). PMA-differentiated THP-1 cells were incubated with Med or CM or NAC-CM. Quantification of ATP in culture supernatant of THP-1 cells by ATP assay (**D**). Representative immunostaining images of ATP in THP-1 cells (scale bar, 30 μm) (**E**). Representative immunoblot analyses of the immature and mature forms of IL-1β, TLR2, Panx1, and cleaved Panx1 in supernatant and cell lysates of THP-1 cells (**F**). Data are presented as the mean ± SD of the representative experiment performed in at least three biological replicates. The *p* values were calculated using one-way ANOVA test. *p < 0.05; **p < 0.01; ***p < 0.001.

To further verify the effects of necrotic TECs on macrophages, we observed whether protecting TECs could inhibit the activation of macrophages. We inhibited the necrosis of TECs by using NAC to protect TECs from cisplatin-induced injury. Flow cytometry analysis showed that the necrosis rate was reduced obviously in NAC-protected TECs (Fig. 6A, B). The protective effects of NAC on TECs were verified by MTT assay (Fig. 6C). ATP secretion was decreased in THP-1 macrophages treated by NAC-protected TECs compared with necrotic TECs (Fig. 6D). Immunofluorescence staining of ATP showed the increased intracellular ATP in THP-1 macrophages treated by NAC-protected TECs (Fig. 6E). Protein levels of TLR2, cleaved Panx1, and mature IL-1β were also downregulated in THP-1 macrophages treated by NAC-CM (Fig. 6F). Therefore, our findings further prove that DAMPs from necrotic TECs can activate TLR2/Panx1/NLRP3 axis in macrophages.

### NAC treatment decreases the activation of TLR2/Panx1/NLRP3 axis in renal tissue

**Fig. 7:**
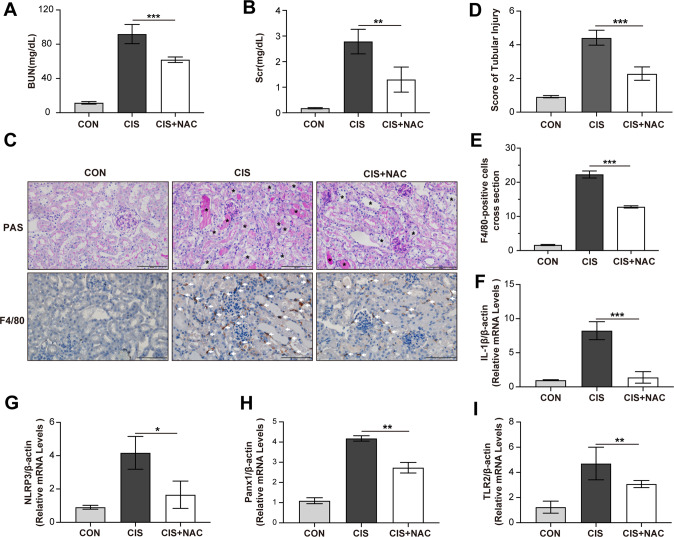
NAC treatment reduces tubular necrosis, decreases macrophage infiltration and inhibits TLR2/Panx1/NLRP3 axis in renal tissue in cisplatin-induced AKI murine model. NAC (500 mg/kg) was given by oral gavage 3 days before cisplatin treatment and 24 h after cisplatin treatment. Serum BUN and creatinine were quantified 3 days after cisplatin treatment (**A**, **B**). Representative PAS staining images and immunohistochemical images F4/80 staining. Asterisk indicates injured renal tubules and white arrow indicates infiltrated macrophages. (scale bar, 100 μm) (**C**). Tubular injury score and quantification of F4/80-positive cells in each group (**D**, **E**). Relative mRNA expressions of IL-1β, NLRP3, Panx1, and TLR2 in renal tissue (**F**–**I**). CON, normal control. Data are presented as the mean ± SD. *n* = 6 at each group The *p* values were calculated using one-way ANOVA test. *p < 0.05; **p < 0.01; ***p < 0.001.

Based on our findings in vitro, we further observed whether protecting TECs could affect renal TLR2/Panx1/NLRP3 axis in AKI murine model. Our previous study has proved that NAC can protect TECs from cisplatin-induced injury [22]. Compared with cisplatin group, cisplatin plus NAC group showed decreased creatinine and BUN levels (Fig. 7A, B). The score of tubular damage was also reduced in cisplatin plus NAC group (Fig. 7C, D). The mRNA level of KIM-1 and NGAL, which are biomarkers of acute tubule injury, were decreased in cisplatin plus NAC group (Supplementary Fig. 6). Less macrophage infiltration could be detected in the renal tissue in cisplatin plus NAC group (Fig. 7C, E). The mRNA levels of TLR2, Panx1, NLRP3, and IL-1β in the renal tissue were also downregulated in cisplatin plus NAC group (Fig. 7F–I). Therefore, results in vivo further confirm our findings in vitro that protecting TECs from necrosis could decrease the activation of TLR2/Panx1/NLRP3 axis.

## Discussion

It is well established that necrotic TECs-induced activation of macrophages have an important role in the early stage of AKI. However, the molecular mechanisms involved in the activation process are unclear. It has been suggested that NLRP3 inflammasome activation in macrophages could play a role, but the pathophysiological link between necrotic TECs and NLRP3 inflammasome activation has not been fully elaborated. In this study, we reveal the role of TLR2 in necrotic TECs-induced NLRP3 inflammasome activation in macrophages during AKI and identify TLR2/caspase-5/Panx1 axis as a novel mechanism for NLRP3 inflammasome activation in macrophages.

In the early stage of AKI, necrotic TECs can create a DAMP-rich interstitial microenvironment. This microenvironment can promote macrophages to polarize towards proinflammatory phenotype and full activation of the proinflammatory macrophages [23]. NLRP3 inflammasome activation plays an important role by producing IL-1β in the process of macrophage polarization and activation [24]. We confirmed the increased infiltration of macrophages accompanies tubular necrosis in renal tissue in cisplatin-induced AKI murine model. Necrotic TECs could stimulate PMA-differentiated THP-1 macrophages to secrete IL-1β, which was decreased in both NLRP3-def and ASC-def THP-1 macrophages. Our findings are consistent with previous studies that necrotic cells can release DAMPs to activate NLRP3 inflammasome in macrophages. Iyer et al. demonstrated that necrotic cells can release mitochondria to activate NLRP3 inflammasome in macrophages [25]. In a contrast-induced AKI study, Lau et al. found that DAMPs from contrast-injured TECs could induce IL-1β released from THP-1 macrophages [26]. However, there is still a lack of comprehensive understanding of the mechanism of how DAMPs activate NLRP3 inflammasome in macrophages during AKI.

As NLRP3 is an intracellular PRR, DAMPs from necrotic cells cannot interact with NLRP3 directly [27]. Rather, NLRP3 can recognize the cell stress induced by DAMPs stimulation [28]. Increased intracellular ROS is one of the best-studied cell stress causing NLRP3 activation [29]. Macrophages under stimulation can secrete endogenous ATP to autocrinally activate P2RX7, which triggers ROS production [29]. The endogenous ATP is mainly secreted via Panx1 channel, a large-pore channel in the cell membrane [21]. Various extracellular stimuli can cause ATP release and subsequent NLRP3 inflammasome activation. Riteau et al. showed that uric acid, silica, or alum crystals could induce ATP release and subsequent purinergic signaling to activate NLRP3 inflammasome in macrophages [30]. Piccini et al. found that the autocrine stimulation of P2RX7 by the released ATP is responsible for NLRP3 activation in monocytes stimulated by various extracellular TLR agonizts [31]. We detected the upregulated expression of both Panx1 and P2RX7 in macrophages stimulated by necrotic TECs. The extracellular level of ATP was increased in macrophages after stimulation. We also showed that blocking Panx1 or antagonizing P2RX7 could inhibit NLRP3 inflammasome activation in macrophages stimulated by necrotic TECs. Therefore, upregulated Panx1 and subsequent ATP release might be key events for necrotic TECs-induced NLRP3 inflammasome activation in macrophages.

Membranous TLRs can bind with DAMPs directly. The activation of NLRP3 inflammasome has been suggested to be initiated by TLRs [28]. Of various TLRs, TLR2 is the major receptor to sense danger signals from necrotic cells [13]. Previous studies have recognized that necrotic cells depend on TLR2 signaling to activate NF-κB in macrophages [14]. NF-κB signaling can modulate NLRP3 inflammasome activation by upregulating the expressions of NLRP3 and pro-IL-1β [15]. Therefore, necrotic cells utilize TLR2/NF-κB pathway to prepare for NLRP3 inflammasome activation. We confirmed the upregulation of TLR2 in macrophages stimulated by necrotic TECs. But we also noticed that TLR2 knockdown could decrease the production of IL-1β in PMA-differentiated THP-1 cells. Our findings agree with studies showing that TLR2 signaling can deliver signals to activate NLRP3 and trigger assembly of the NLRP3 inflammasome complex independent of NF-κB pathway. Lin et al. discovered that TLRs can directly regulate activation of NLRP3 through IL-1 receptor-associated kinase [16]. Wu et al. showed that TLR2 signaling could induce the cleavage of pro-caspase-1 and NLRP3 activation in allergic airway inflammation murine model [17]. Based on these findings, it is necessary to elucidate the exact role of TLR2 in necrotic TECs-induced NLRP3 inflammasome activation in macrophages.

We found that TLR2 knockdown could decrease ATP released from macrophages. It has been observed that ATP release from macrophages occurs in response to TLR signaling. Ren et al. revealed that TLR agonizts like LPS and Pam3CSK4 could induce ATP secretion from macrophages [32]. On the mechanism of TLR-induce ATP secretion, Cohen et al. demonstrated that Panx1 is the major conduit by which ATP is released from TLR-stimulated macrophages [33]. Ren et al. considered that ATP can be secreted from macrophages through TLR-dependent intracellular calcium mobilization [32]. We confirmed that TLR2 knockdown could reduce the protein level of cleaved Panx1 and the secretion of ATP from macrophages. A previous study revealed that TLR2 agonist can cause activation of Panx1 and release of ATP to activate NLRP3 inflammasome in THP-1 cells [20]. However, they did not delineate the mechanism of TLR2-induced Panx1 activation.

As a channel formation protein, Panx1 can be activated by proteolytic cleavage, which is initiated by caspases. Yang et al. found that intracellular LPS can induce the activation of Panx1 through caspase-11 [34]. Mouse caspase-11 and its human ortholog caspase-4/5 are identified as a sensor of intracellular pathogen infection [35]. However, recent studies showed that noninfectious factors can also upregulate caspase-11 expression, especially in kidney disease [36, 37]. Upregulated caspase-11 could cleave Panx1 to facilitate ATP release and NLRP3 inflammasome activation in renal tubular cells [37]. We found that both the mRNA and protein levels of caspase-5 could be upregulated in macrophages stimulated by necrotic TECs. Knocking down caspase-5 could downregulate the protein level of cleaved Panx1 and reduced the secretion of IL-1β by macrophages. TLR2 siRNA treatment could decrease the protein level of caspase-5 in macrophages. Flis et al. found that oesophageal adenocarcinoma cells can release HMGB1 to activate TLR2, which can upregulate caspase-11 expression [38]. It has been found that caspase-11 or caspase-4/5 can promote IL-1β maturation and secretion by triggering the activation of the canonical NLRP3 inflammasome, which is considered as a non-canonical inflammasome pathway [11]. Therefore, our study reports the non-canonical activation of NLRP3 inflammasome in macrophages by necrotic TECs. The mechanism of TLR2-induced caspase-11 or caspase-4/5 activation deserves further studies.

As necrotic TECs release DAMPs to stimulate TLR2, we further investigated the effects of reducing necrosis by protecting TECs on TLR2/Panx1/NLRP3 axis activation in macrophages. By using NAC to protect TECs, we detected significantly decreased necrosis in TECs treated by cisplatin. The protein levels of TLR2, cleaved Panx1, and IL-1β in macrophages were decreased by CM from protected TECs compared with CM from necrotic TECs. In cisplatin-induced AKI murine model, we also proved that NAC could decrease the infiltration of macrophages and reduce the mRNA levels of TLR2, Panx1, and IL-1β in the renal tissue. From another perspective, our results support that necrotic TECs can release DAMPs to activate TLR2/Panx1/NLRP3 axis in proinflammatory macrophages. Previous studies have uncovered several candidate molecules from necrotic cells to activate proinflammatory macrophages [39–41]. Further studies will be required to clarify which DAMPs from necrotic TECs activate TLR2/Panx1/NLRP3 axis in macrophages.

It has been acknowledged that DAMPs to activate TLR2 are accumulated in renal tissue during AKI [42]. TLR2 has been found to express constitutively within the kidney and participate in the pathogenesis of AKI [43]. Previous studies indicate that the absence of TLR2 can decrease renal inflammation and reduce renal injury in both ischemia/reperfusion and cisplatin-induced AKI murine models [42, 44]. By using chimeric mice, Leemans et al. considered that TLR2 on renal parenchymal cells rather than TLR2 on circulating cells played a major role in the induction of inflammation and renal injury in the early stage of AKI [42]. But they could not exclude that TLR2 on circulating cells might also participate in renal injury. In an acute myocardial ischemia-reperfusion injury murine model, Arslan et al. detected that TLR2 in leukocytes mediated myocardial injury rather than TLR2 in parenchymal cells [45]. All these studies use bone marrow chimaera mice to elucidate the role of TLR2 in the renal parenchyma versus haematopoietic cells, which may not be precise enough [11]. Using cell specific conditional TLR2 knockout mice will be necessary to clarify the role of TLR2 in macrophages in the early stage of AKI.

**Fig. 8:**
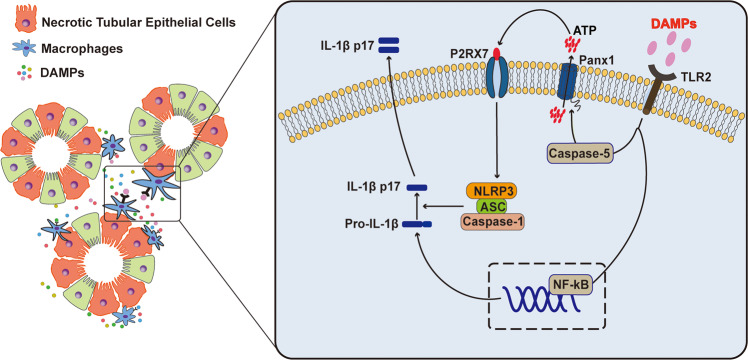
Schematic illustrating the proposed mechanism by which danger signals from necrotic TECs activate NLRP3 inflammasome in macrophages. We propose that danger signals from necrotic TECs can bind with TLR2 in macrophages, which upregulates caspase-5 and trigger Panx1 cleavage. Intracellular ATP released from macrophages through Panx1 channel can stimulate P2RX7 and cause the activation of NLRP3 inflammasome to produce IL-1β.

In conclusion, our work has identified TLR2 as a critical receptor for DAMPs from necrotic TECs to cause NLRP3 inflammasome activation in macrophage during AKI. Experimental evidence suggests that DAMPs from necrotic TECs promote NLRP3 inflammasome activation in macrophages through TLR2/caspase-5/Panx1 axis (Fig. 8). These results suggest that TLR2/caspase-5/Panx1 axis could be a novel approach to modulate NLRP3 inflammasome activation in macrophages during necroinflammation.

## Material and methods

### Animal

Male C57BL/6 mice (8–12 weeks old, 20–25 g, Guangdong Medical Laboratory Animal Center, Guangdong, China) was used in this study. All animal experiments underwent the ethical review and were approved by the Institutional Animal Care and Use Committee of Southern Medical University. The experiment protocol was conducted according to the National Institute of Health (NIH) Guide for the Care and Use of Laboratory Animals, as well as with the ARRIVE guidelines [46]. Mice were treated according to the guidelines of the institutional animal care and use committee and were maintained in a laminar-flow, pathogen-free atmosphere. Mice were anesthetized by intraperitoneal administration of 1% pentobarbital sodium (10 μl/g) and were sacrificed by intraperitoneal administration of pentobarbital sodium (150 mg/kg). All the dead animals were disposed of by the Laboratory Animal Center. Mice were randomly assigned to either control or experimental groups at the beginning of each experiment. Blinding or sample size estimation tests were not applied and no animals were excluded from analysis.

### Cisplatin-induced AKI models

For the cisplatin-induced AKI murine models, mice were treated with vehicle or 25 mg/kg dose of cisplatin via intraperitoneal injection and sacrificed after 72 h. For NAC (N-Acetyl-L-Cysteine) treatment, the vehicle (0.5 ml saline) or NAC (500 mg/kg/day, dissolved in 0.5 ml saline) were administered orally by gavage every day from 3 days prior to the first dose of cisplatin treatment and 24 h after cisplatin treatment.

### Creatinine and BUN measurement

Blood samples of C57BL/6 mice were centrifuged at 3.000 rpm/min for 10 min at 4 °C. The serum creatinine and urea nitrogen (BUN) were assessed on an auto-analyzer (Beckman DXC600, Brea, CA, USA).

### Histopathology

For renal histology, renal tissues were immersed and fixed in 4% paraformaldehyde (PFA) for 24 h and then embedded in paraffin. Renal sections (2 μm) were stained with periodic-acid Schiff (PAS). Sections were analyzed for the acute tubular injury including tubular cell necrosis, tubular dilation, intratubular detachment, and evaluated in a blinded manner by a nephrologist. Acute tubular necrosis severity was semi-quantified using the following scoring system: 0 = no abnormalities; 1 = <10% of the tubules; 2 = 11% to 25%; 3 = 25% to 45%; 4 = 46% to 75%; 5 = ≥76%.

### Immunohistopathology

For immunohistochemical microscopy, renal tissue was perfusion-fixed in 4% PFA and embedded in paraffin, and 2-μm paraffin sections were mounted on slides. After deparaffinization and dehydration, sections were subjected to antigen retrieval and blocked with 1% BSA before incubation in primary antibodies against F4/80(1:100, Abcam) overnight at 4 °C and then incubated with secondary antibodies conjugated with peroxidase. Finally, staining was developed using a DAB kit (ZSGB-BIO, Beijing, China) followed by hematoxylin counter-staining.

### Cell culture and treatment

NRK-52E cells were cultured as previously described [22]. The THP-1 monocytic cell line was purchased from the American Type Culture Collection (Manassas, USA) and cultured 1.0 × 10^6^ cells/ml in RPMI 1640 supplemented with glutamine, 10% fetal bovine serum (FBS) (GIBICO, USA), 100 U/ml penicillin and 100 U/ml streptomycin at 37 °C under a 5% CO_2_ atmosphere. Medium and reagents were purchased from GIBCO/Thermo Fisher Scientific (Waltham, USA). THP-1 cells were differentiated with 100 ng/ml PMA (Sigma-Aldrich) for 48 h, washed with PBS, and then cultured for 24 h in fresh medium supplemented with 2% FBS before stimulation. NLRP3-deficient THP-1 (Def-NLRP3-THP-1) and ASC-deficient THP-1 (Def-ASC-THP-1) cell lines were purchased from Invivogen (San Diego, USA). All cells used in this study were identified by short tandem repeat authentication and excluded to mycoplasma contamination.

For in vitro cisplatin-induced AKI models, NRK-52E cells were seeded at 1.0 × 10^4^ cells/cm^2^. Twenty-four hours later, the cells were treated with DMEM or 20 μM cisplatin for 6 h. The medium was then removed, and the cells were washed with PBS twice. Finally, the cells were cultured with DMEM containing 2% FBS for 24 h to 72 h. To evaluate the effect of NAC treatment, cells were pre-incubated with 50 mM NAC for 1 h before the treatment of cisplatin. The dose level and schedule were based on previous studies [22].

After differentiation, THP-1 cells were treated with LPS (Sigma-Aldrich), ATP (Promega, Beijing, China), Z-VAD (Biovision, Dalian, China), Z-YVAD (Biovision), suramin (Cayman, Michigan, USA), and probenecid (Sigma-Aldrich) before or after adding conditioned medium. The concentration of each compound was stated in figure legends.

### Generation of conditioned medium

To generate the conditioned medium (CM) from cisplatin-injured NRK-52E cells, 1 × 10^4^ NRK-52E cells/cm^2^ were cultured in culture plates, and after 6 h treatment with 20 μM cisplatin (Sigma-Aldrich), the culture medium was changed to DMEM containing 2% FBS, which the cells were cultured in for 48 h. The medium was then harvested and centrifuged at 4000 rpm/min for 15 min to remove cell debris and the supernatant was stored at −80 °C until use. Control medium (Med) was collected from the medium of cultured normal NRK-52E cells. To generate the NAC-protected conditioned medium (NAC-CM), NRK-52E cells were pre-incubated with 50 mM NAC for 1 h before being treated with cisplatin. Then the culture medium was changed to 2% FBS DMEM for 48 h and medium was harvested.

### Cell counts and MTT

NRK-52E cells were treated with cisplatin as previously described. Viable cells were counted by trypan blue dye exclusion (Sigma-Aldrich). Cell viability was also evaluated by MTT assay (Sigma-Aldrich).

### ELISA assays

THP-1 Cell culture supernatant level of IL-1β was quantified using Quantikine for human IL-1β (R&D Systems, Minneapolis, MN, USA) according to the manufacturer’s recommendations.

### ATP measurement

To study ATP released from THP-1 cells, the supernatant or the cell lysates of CM-treated THP-1 cells were subjected to the luciferin-based ENLITEN ATP Assay (Promega), according to the manufacturer’s instructions.

### Flow cytometry analysis

Necrotic NRK52-E cells was detected by using FITC Annexin V/7-AAD Viability Staining Solution (Biolegend) according to the manufacturer’s instruction. Stained cells were assessed necrotic activity using a FACSCanto II Flow cytometry (Becton Dickinson, San Jose, CA, USA). Data were analyzed with FlowJo software (Tree Star Inc., Ashland, OR, USA).

### Immunoblot analysis

Proteins from THP-1 cells were extracted by RIPA lysis buffer (Cell Signaling Technology, Danvers, MA, USA) with phenylmethanesulfonyl fluoride (PMSF) (Cell Signaling Technology). For supernatant protein immunoblot, 0.5 ml supernatant was collected and mixed with 0.5 ml methanol and 0.125 ml chloroform to precipitate all proteinaceous materials. The samples were then centrifuged at 12.000 rpm/min for 10 min, and the top layer was removed. Then 0.5 ml of methanol was added to resuspend the proteins and were centrifuged for 10 min at 12.000 rpm/min. The remaining supernatant was aspirated, and the pellet was resuspended in 100 μl RIPA lysis buffer with PMSF after being dried at 37 °C for 5 min. We then measured protein concentration in the pellet using the BCA assay kit (Pierce, Rockford, IL, USA). Afterwards, the pellet was resuspended in 25 μl of 5 × SDS loading buffer and heated at 95 °C for 5 min until the pellet was dissolved. Samples containing 20 μg of protein were loaded onto gels and separated by SDS-PAGE, followed by blotting on a 0.22 μm or 0.45 μm PVDF membrane (Millipore). Immunoblotting was performed using specific antibodies overnight at 4 °C using the following antibodies: primary antibodies rabbit polyclonal anti-human IL-1β (1:200, Santa Cruz Biotechnology, Dallas, TX, USA), rabbit polyclonal anti-human caspase-1 (1:200, Santa Cruz Biotechnology), rabbit polyclonal anti-human Pannexin-1 (1:500, GeneTex, CA, USA), rabbit polyclonal anti-human P2RX7 (1:200, GeneTex), rabbit polyclonal anti-TLR2 (1:500, Abcam, Cambridge, USA), Mouse monoclonal anti-TLR4 (1:500, Abcam), anti-caspase-5 (1:500, Cell Signaling Technology). Then, the membranes were incubated with HRP-conjugated Affinipure Goat Anti-Rabbit IgG (H + L) (1:3000, Proteintech) or HRP-conjugated Affinipure Goat Anti-Mouse IgG (1:3000, Proteintech). Immunoreactive bands were visualized by an ECL-based detection method.

### Quantitative RT-PCR

Total RNA from kidney tissues and THP-1 cells was extracted using RNAIso Plus (Takara, Kusatsu, Japan), and 2 μg of total RNA was used for reverse transcription using the PrimeScriptRT reagent kit (Takara). Quantitative real-time polymerase chain reaction (qRT-PCR) was performed using a SYBR Green Supermix kit (Takara). Relative expression was normalized to the expression levels of β-actin. The primer sequences used for PCR are shown in Table 1.

**Table 1:** Primers used for real-time PCR.

Gene	Forward primer	Reverse primer
Human NLRP3	CCACAAGATCGTGAGAAAACCC	CGGTCCTATGTGCTCGTCA
Human NLRP1	GTGAAACCAGGAAGGAACACCA	AAGCCCTCTCTGGCTTCATTG
Human AIM2	GAAGCCGTCCAGAAGTGTCA	TGCTTATCAACTCCTGATCCCT
Human NLRC4	TGCATCATTGAAGGGGAATCTG	GATTGTGCCAGGTATATCCAGG
Human CASPASE-1	TTTCCGCAAGGTTCGATTTTCA	GGCATCTGCGCTCTACCATC
Human CASPASE-4	AAGAGAAGCAACGTATGGCAGGAC	GGACAAAGCTTGAGGGCATCTGTA
Human CASPASE-5	GGTGAAAAACATGGGGAACTC	TGAAGAACAGAAAGCAATGAAGT
Human P2RX7	TACCAGCGGAAAGAGCCTGT	CGGTCTGAATTCCTTTGCTCTG
Human P2RX4	TTGGCAGAAGTGGAAATGGAGT	TTTCCTCCTGAGCTGGTATCACA
Human PANX1	CCATGGCCATCGCTCAACT	CTGTGTACCAATCGAGATCTCCTG
Human TLR1	TTCAAACGTGAAGCTACAGGG	CCGAACACATCGCTGACAACT
Human TLR2	ATCCTCCAATCAGGCTTCTCT	GGACAGGTCAAGGCTTTTTACA
Human TLR4	TGGCCCTAAACCACACAGAA	TGTCTGGATTTCACACCTGGAT
Human TLR6	TTCTCCGACGGAAATGAATTTGC	CAGCGGTAGGTCTTTTGGAAC
Human β-actin	CATGTACGTTGCTATCCAGGC	CTCCTTAATGTCACGCACGAT
Mouse NLRP3	ATTACCCGCCCGAGAAAGG	CATGAGTGTGGCTAGATCCAAG
Mouse IL-1β	TTCAGGCAGGCAGTATCACTC	GAAGGTCCACGGGAAAGACAC
Mouse KIM-1	ACATATCGTGGAATCACAACGAC	ACTGCTCTTCTGATAGGTGACA
Mouse NGAL	ACAACCAGTTCGCCATGGTA	AGCTCCTTGGTTCTTCCATACAG
Mouse PANX1	CTTCACTCTCGGACGAGTTTC	GGAATCAGCAGAGCATACACAAT
Mouse TLR2	CTCTTCAGCAAACGCTGTTCT	GGCGTCTCCCTCTATTGTATTG
Mouse β-actin	GTGACGTTGACATCCGTAAAGA	GCCGGACTCATCGTACTCC

### Immunofluorescence

For detection of intracellular ATP, cells were labeled with 1 μM quinacrine (Sigma-Aldrich) in Krebs-Ringer solution for 1 h at 37 °C and then were fixed with 2% paraformaldehyde for 15 min at room temperature. Finally, the cells were stained with DAPI (Sigma-Aldrich) for 10 min.

For ASC staining, THP-1 cells were fixed with 4% paraformaldehyde for 15 min at room temperature and incubated with PBS solution containing 0.3% Triton X-100 for 15 min at room temperature. Then cells were incubated with rabbit anti-human ASC antibody (1:100, AdipoGen, San Diego, USA) overnight at 4 °C. Then immunofluorescence was performed using Alexa Fluor 594-goat anti-rabbit antibody (1:50, Proteintech). Cells were stained with DAPI for 15 min at room temperature. Fluorescence imaging was performed with confocal microscopy (Olympus, Shanghai, China). The negative control was carried out with the isotype control antibody.

### RNA interference

Small interference RNA (siRNA) specific against the human TLR2 gene or human caspase-5 gene and scrambled RNA were designed and synthesized by GenePharma (Suzhou, China). THP-1 cells were transfected with control scramble siRNA or 20 nM TLR2 siRNA or 20 nM caspase-5 siRNA using Lipofectamine 3000 (Invitrogen, Shanghai, China) for 36 h. The efficiency of siRNA transfection was detected by WB for the protein expression of TLR2 and caspase-5.

### PPI networks

For this study, the online database STRING (Search Tool for the Retrieval of Interacting Genes/Proteins) was used to reveal the protein–protein interaction networks among the target proteins (http://string-db.org). The experimental and theoretical interaction networks of caspase-5 were provided with unique coverage and ease of access by this tool. The data were shown in Table 2.

**Table 2:** Caspase-5 functional partners depicted by STRING.

Protein	Function	Combined_Score
TLR2	Toll-like receptor 2	0.459
PANX1	Pannexin-1	0.527
P2RX7	P2X purinoceptor 7	0.507
NLRP3	NACHT, LRR and PYD domains-containing protein 3	0.985
PYCARD(ASC)	Apoptosis-associated speck-like protein containing a CARD	0.996
CASP-1	Caspase-1	0.937
IL1B	Interleukin-1 beta	0.81

### Statistical analysis

All statistical analysis was performed using GraphPad Prism 9.0 (GraphPad Software Inc., San Diego, CA, USA). Data are expressed as means ± SD of at least three independent experiments. Differences between two groups were performed by the Student’s *t* test and multiple groups were evaluated by one-way ANOVA, followed by Tukey’s multiple-range test. *P* values < 0.05 were considered statistically significant.

## Supplementary information

Supplementary table

Supplementary figure legend

Supplementary Fig.1

Supplementary Fig.2

Supplementary Fig.3

Supplementary Fig.4

Supplementary Fig.5

Supplementary Fig.6

Supplementary Fig.7

## Data Availability

The data generated or analyzed during this study are included in this published article and its Supplementary Information files.
