# Supplementary material for: TLR2/caspase-5/Panx1 pathway mediates necrosis-induced NLRP3 inflammasome activation in macrophages during acute kidney injury
Source: Cell Death Discov. 2022 Apr 26;8:232. doi: 10.1038/s41420-022-01032-2 (PMC9042857; doi:10.1038/s41420-022-01032-2)
Supplement: Supplementary file 1 — Supplementary table [file 41420_2022_1032_MOESM1_ESM.docx]

**Supplementary Table.1**

| **Antibody Name** | **Vendor** | **Cat Num** |
| --- | --- | --- |
| IL-1beta (H-153) antibody | Santa Cruz Biotechnology | sc-7884 |
| Caspase-1 p10 (M-20) antibody | Santa Cruz Biotechnology | sc-514 |
| Rabbit anti-Pannexin-1 Polyclonal Antibody | GeneTex | GTX46955 |
| Rabbit anti-P2RX7 Polyclonal Antibody | GeneTex | GTX104288S |
| Rabbit anti-TLR2 Polyclonal Antibody | Abcam | ab24192 |
| Mouse anti-TLR4 Monoclonal Antibody | Abcam | ab22048 |
| Caspase-5 Rabbit mAb antibody | Cell Signaling Technology | 46680 |
| Anti-F4/80 Antibody | Abcam | ab6640 |
| Rabbit anti-ASC Polyclonal Antibody | AdipoGen | AG-25B-0006 |
| Beta Actin antibody | Proteintech | 60008-1-Ig |
| Goat anti-mouse IgG (H+L), HRP conjugate antibody | Proteintech | SA00001-1 |
| Goat anti-Rabbit IgG (H+L), HRP conjugate antibody | Proteintech | SA00001-2 |
| CoraLite594 conjugated Goat Anti-Mouse IgG(H+L) antibody | Proteintech | SA00013-3 |
