# Supplementary material for: TLR2/caspase-5/Panx1 pathway mediates necrosis-induced NLRP3 inflammasome activation in macrophages during acute kidney injury
Source: Cell Death Discov. 2022 Apr 26;8:232. doi: 10.1038/s41420-022-01032-2 (PMC9042857; doi:10.1038/s41420-022-01032-2)
Supplement: Supplementary file 2 — Supplementary figure legend [file 41420_2022_1032_MOESM2_ESM.docx]

**Supplementary Figure 1 Detection of renal function in cisplatin-induced AKI murine.**

The C57/BL mice were administered by intraperitoneal injection of cisplatin (25mg/kg) and sacrificed 3 days later. Renal function was evaluated by serum creatinine and BUN (A, B). Values are the mean ± SD; ****p* < 0.001 versus CON group. *n* = 6 mice per group.

**Supplementary Figure 2 Detection of necrosis occurrence in tubular epithelial cell treated by cisplatin.**

NRK-52E cells were treated with 20 μM cisplatin for 6 h. 6 h later, the culture medium was changed to DMEM containing 2% FCS and the cells were cultured for 24 h to 72 h. Cell proliferation assay by cell counts (A). Combined Annexin-V and 7-AAD staining was used to distinguish. Q1: necrotic cells (annexin V-/PI+); Q2: late apoptosis (annexin V+/PI+); Q3: apoptotic cells; (annexin V+/PI-) Q4: live cell (annexin V-/PI-). Representative flow cytometry analysis and summarized results of TECs (B and C). Values are the mean ± SD; **p* < 0.05, ***p* < 0.01, ****p* < 0.001, versus CON group. *n* ≥3 per group.

**Supplementary Figure 3 The pattern diagram of conditioned medium collection**

**Supplementary Figure 4 Caspase inhibitor reduces the IL-1β production in macrophages.**

PMA-differentiated THP-1 cells were pretreated with Z-VAD-FMK (pan-caspase inhibitor) or Z-YVAD-FMK (casapase-1 inhibitor) for 2 h before being incubated with Med or CM for 6 h. Quantification of IL-1β in supernatant by ELISA. Values are the mean ± SD; **p* <0.05 versus CM group. *n*≥3 per group.

**Supplementary Figure 5 TLR4 knock down does not decrease the secretion of ATP by macrophages**

PMA-differentiated THP-1 cells were transfected with TLR4 siRNA for 36 h before being treated with Med or CM. Quantification of ATP in the supernatant of THP-1 cells by ATP assay kit. Values are the mean ± SD; *n* ≥3 per group.

**Supplementary Figure 6 NAC treatment alleviates the renal tubular injury in cisplatin-induced AKI murine model**

NAC (500mg/kg) was given by oral gavage 3 days before cisplatin treatment and 24 h after cisplatin treatment. Relative mRNA expressions of KIM-1 and NGAL in renal tissue. Values are the mean ± SD; **p* < 0.05, ***p* < 0.001. *n* = 6 mice per group.
