# Supplementary figures and images for: TLR2/caspase-5/Panx1 pathway mediates necrosis-induced NLRP3 inflammasome activation in macrophages during acute kidney injury
Source: Cell Death Discov. 2022 Apr 26;8:232. doi: 10.1038/s41420-022-01032-2 (PMC9042857; doi:10.1038/s41420-022-01032-2)

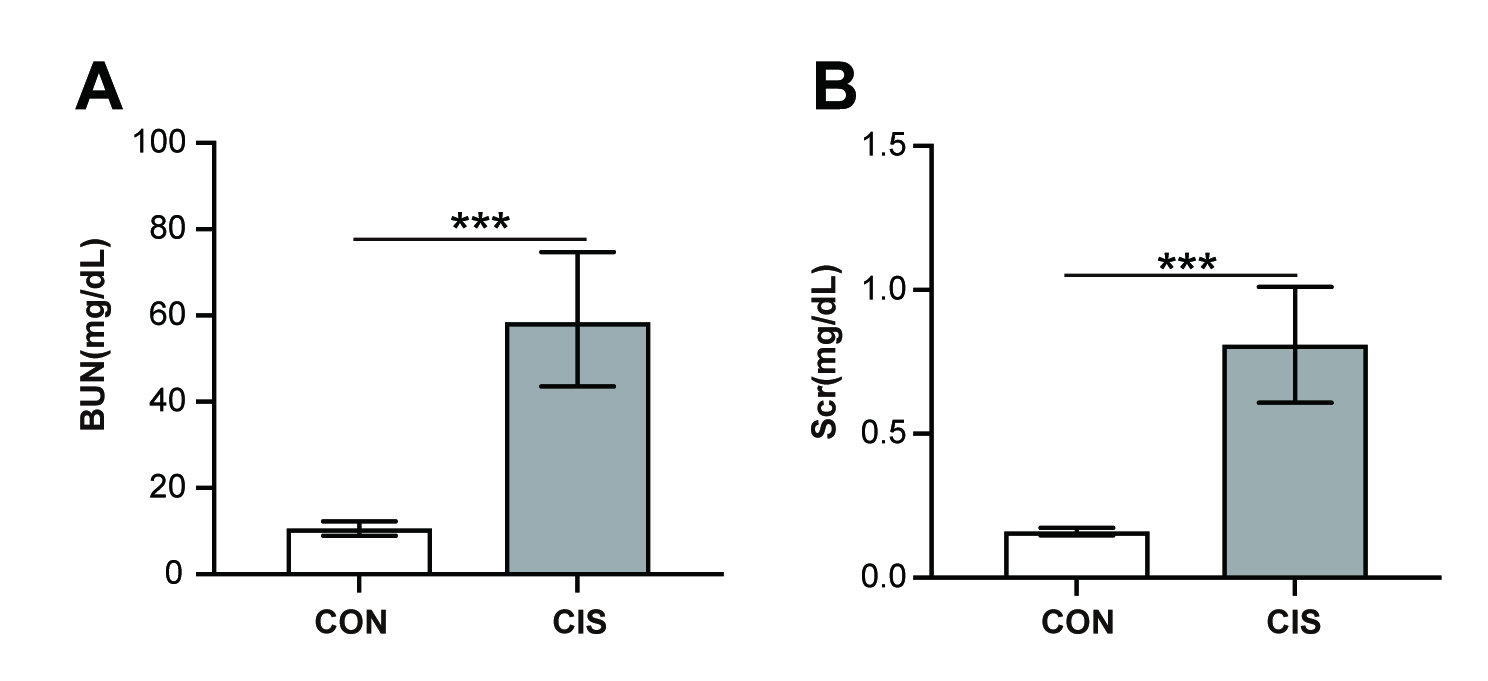

Supplement: Supplementary file 3 — Supplementary Fig.1 [file 41420_2022_1032_MOESM3_ESM.tif]

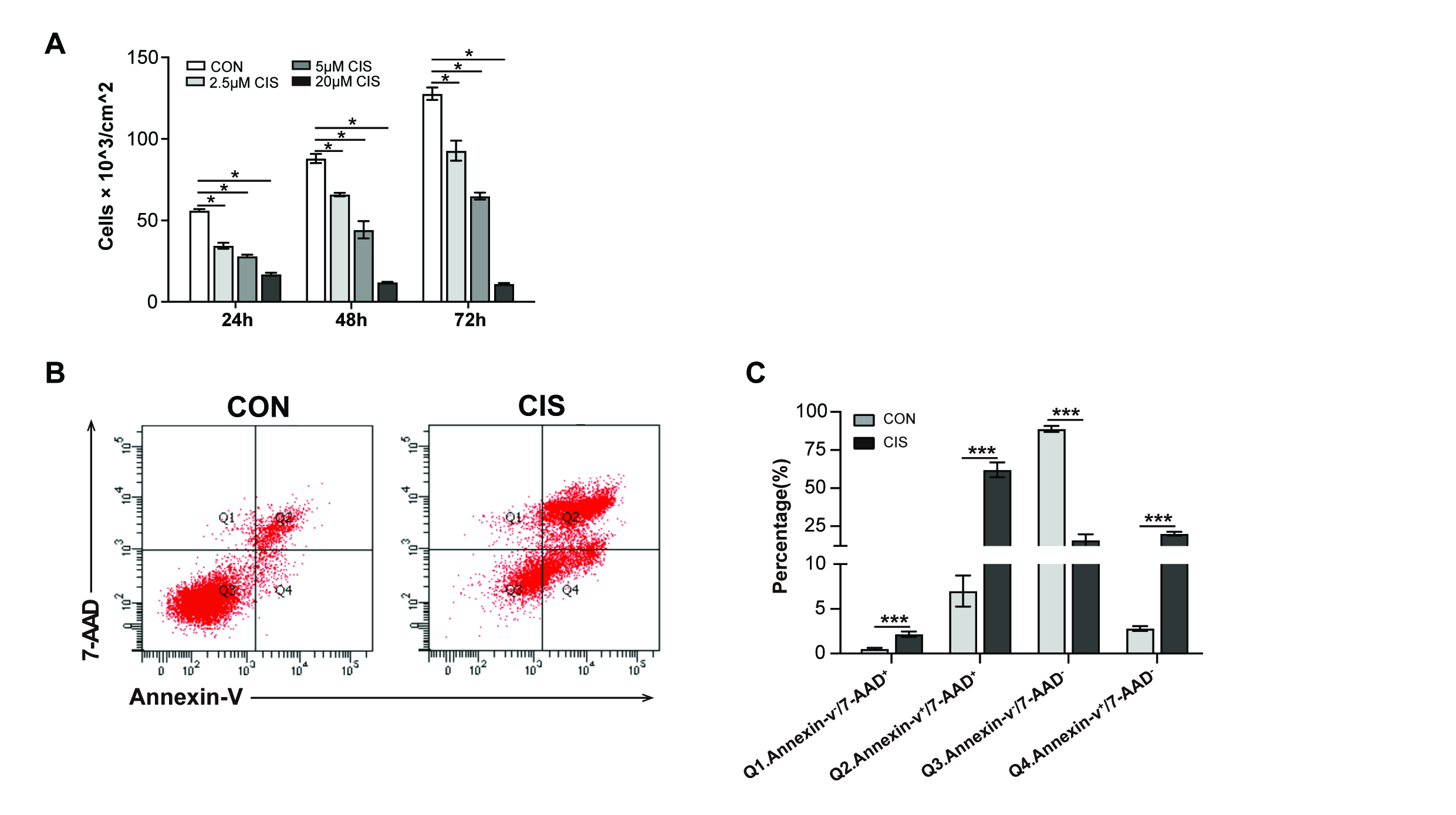

Supplement: Supplementary file 4 — Supplementary Fig.2 [file 41420_2022_1032_MOESM4_ESM.tif]

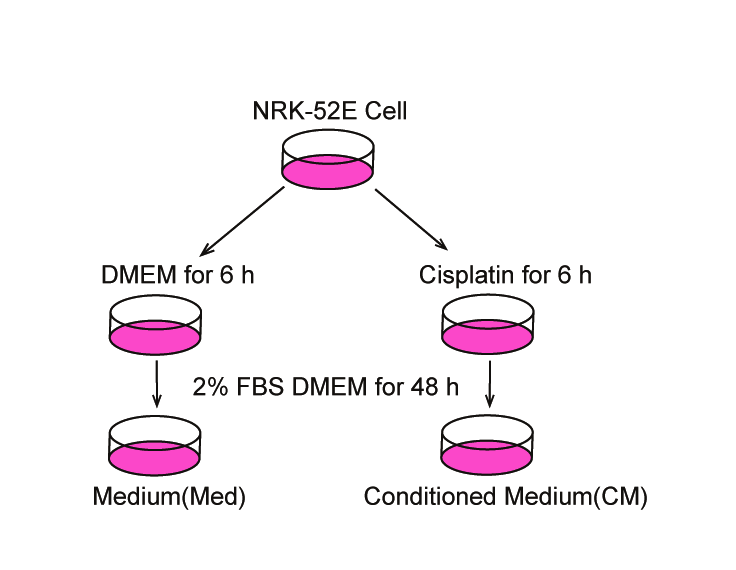

Supplement: Supplementary file 5 — Supplementary Fig.3 [file 41420_2022_1032_MOESM5_ESM.tif]

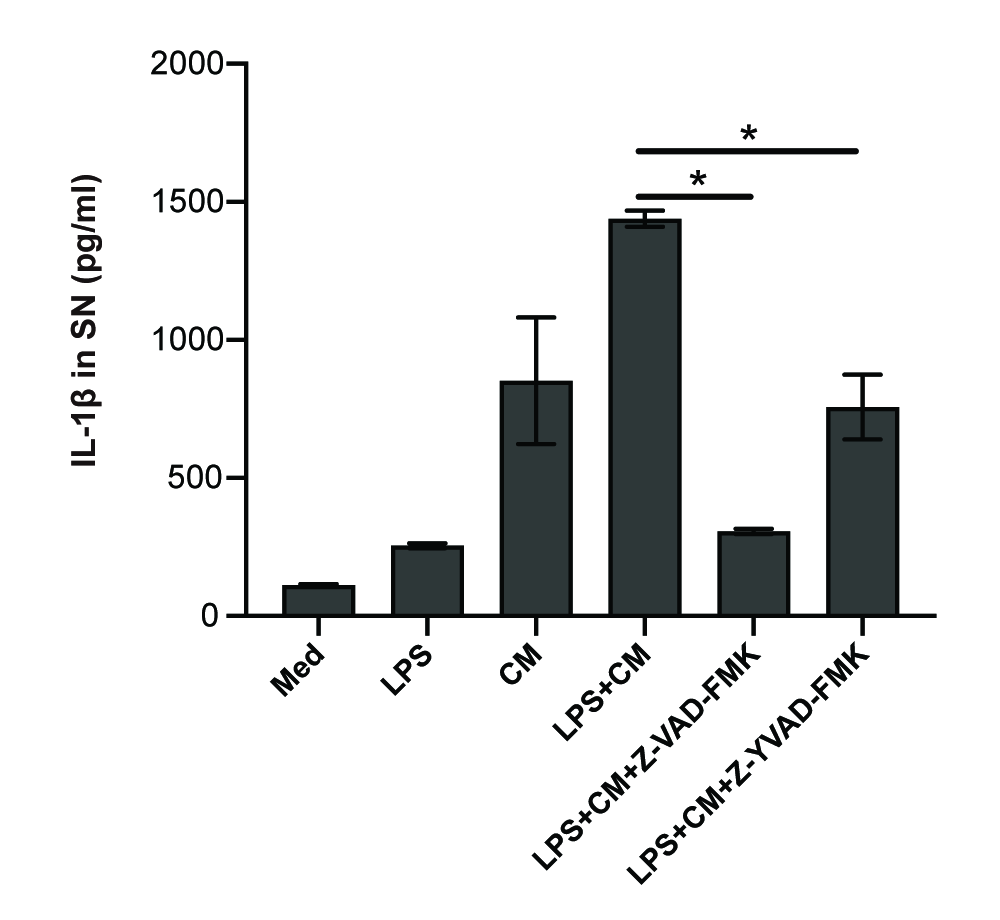

Supplement: Supplementary file 6 — Supplementary Fig.4 [file 41420_2022_1032_MOESM6_ESM.tif]

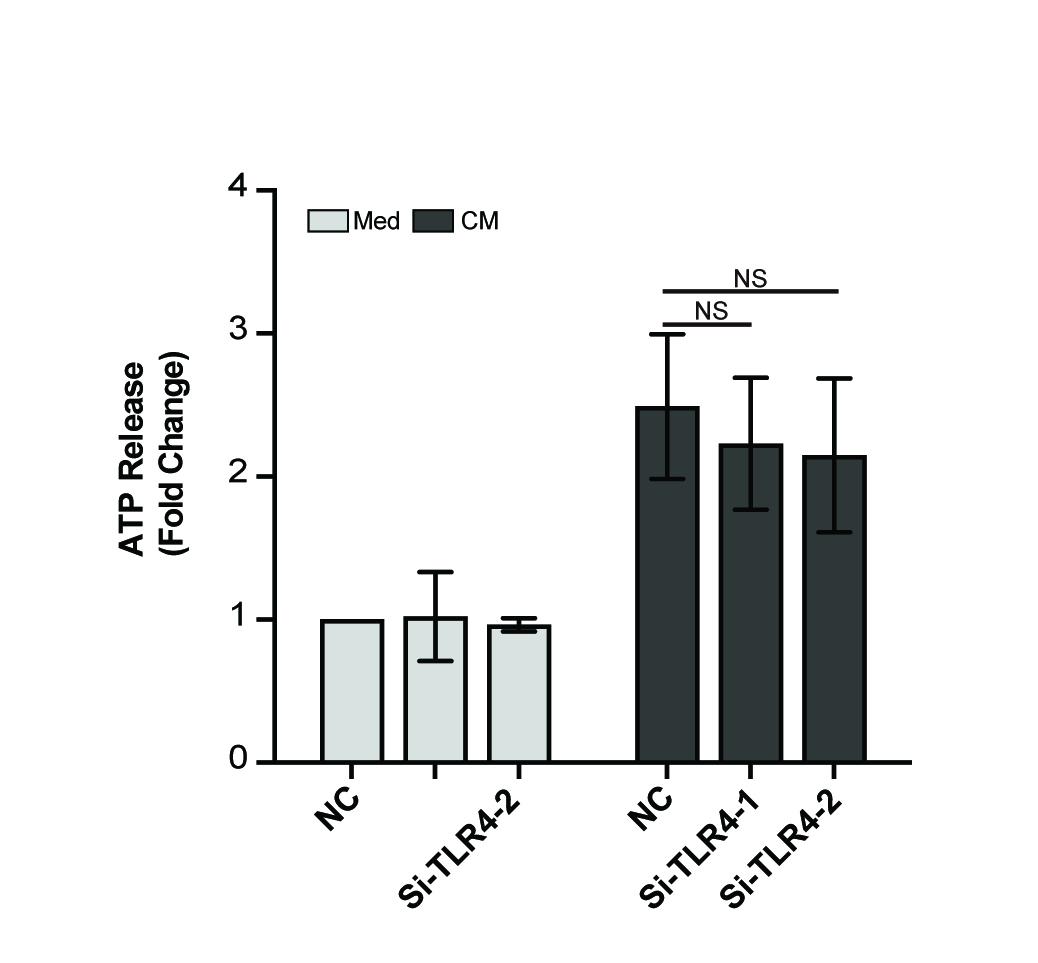

Supplement: Supplementary file 7 — Supplementary Fig.5 [file 41420_2022_1032_MOESM7_ESM.tif]

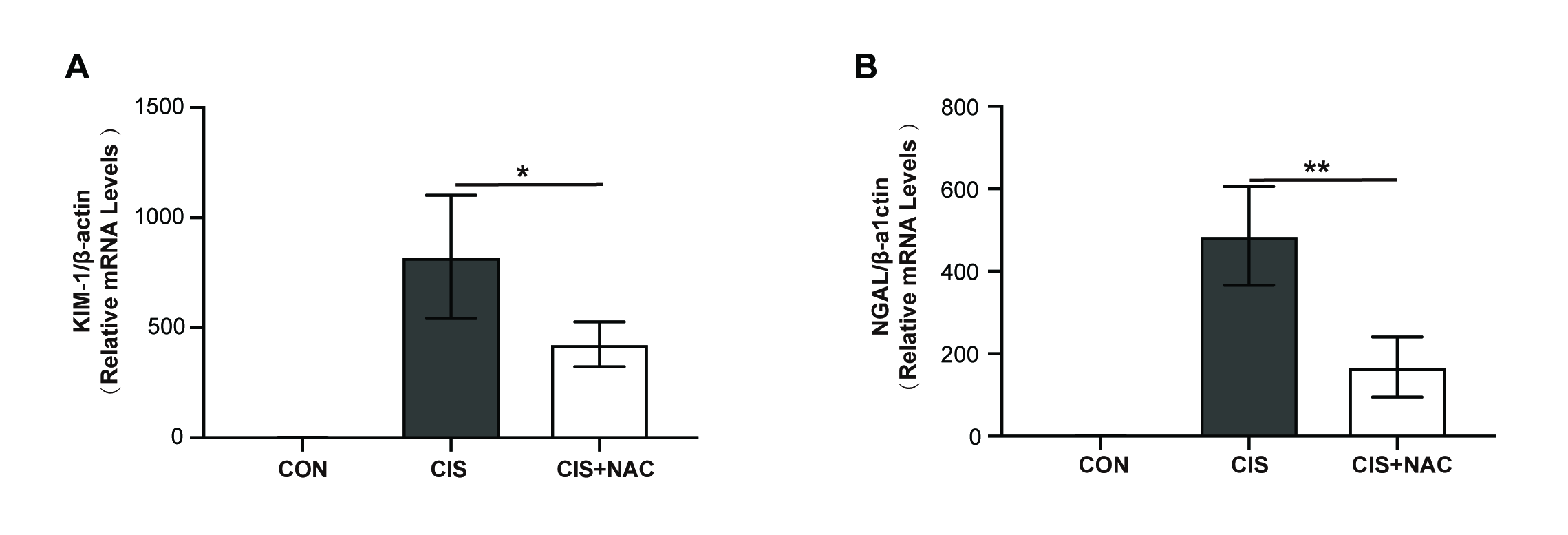

Supplement: Supplementary file 8 — Supplementary Fig.6 [file 41420_2022_1032_MOESM8_ESM.tif]

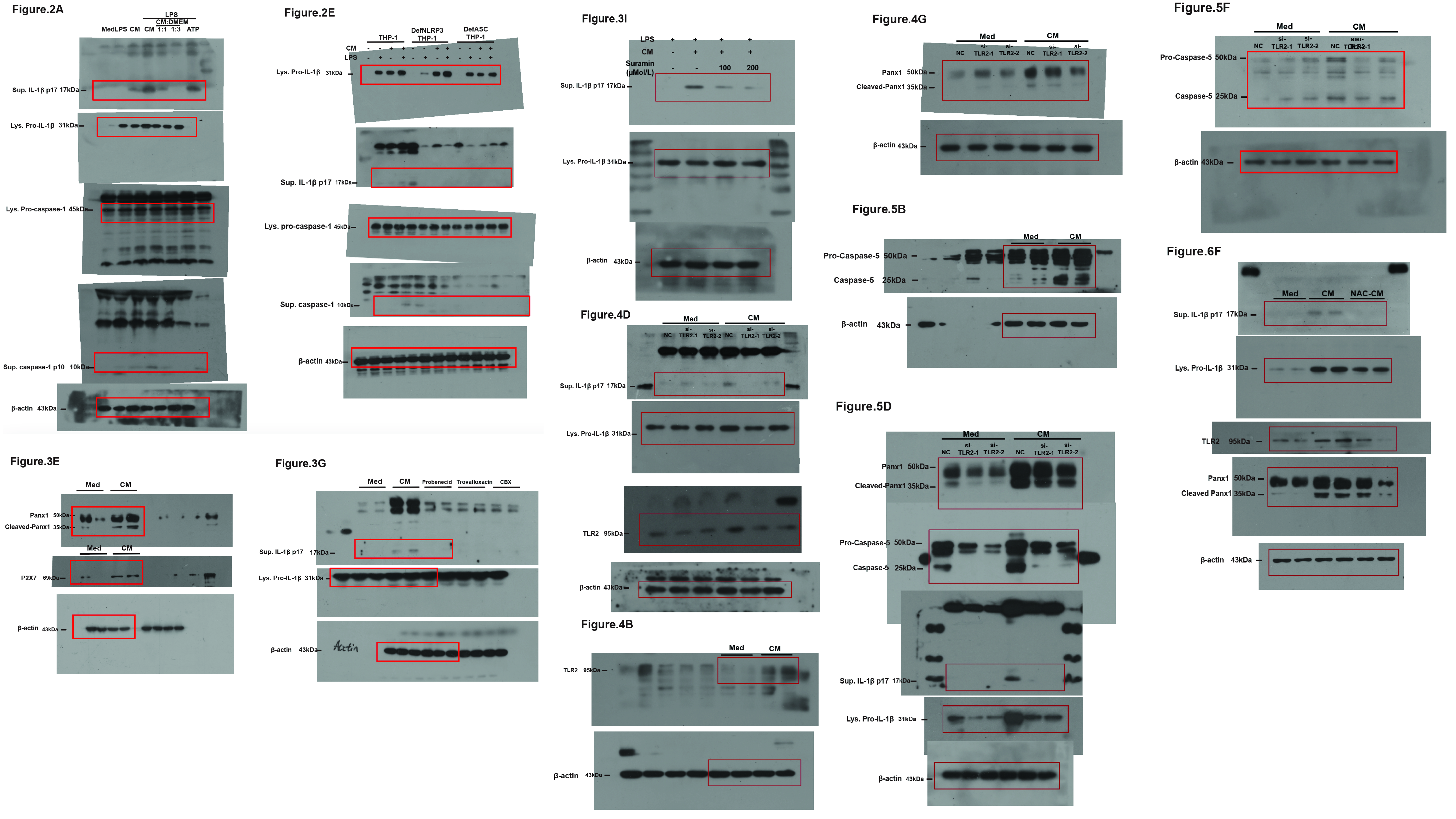

Supplement: Supplementary file 9 — Supplementary Fig.7 [file 41420_2022_1032_MOESM9_ESM.tif]
